# Pre- and Posttherapy Assessment of Intestinal Soluble Mediators in IBD: Where We Stand and Future Perspectives

**DOI:** 10.1155/2013/391473

**Published:** 2013-05-08

**Authors:** F. Scaldaferri, V. Petito, L. Lopetuso, G. Bruno, V. Gerardi, G. Ianiro, A. Sgambato, A. Gasbarrini, G. Cammarota

**Affiliations:** ^1^Department of Internal Medicine, Gastroenterology Division, Catholic University of Sacred Heart, Policlinico A. Gemelli Hospital, Roma, Italy; ^2^Institute of Pathology, Catholic University of Sacred Heart, Rome, Italy

## Abstract

Inflammatory bowel disease (IBD) is a chronic inflammatory condition characterized by an abnormal immune response against food or bacterial antigens in genetically predisposed individuals. Several factors of innate and adaptive immune system take part in the inflammatory process, probably actively contributing in endoscopic and histological healing at molecular level. Although it is difficult to discriminate whether they are primary factors in determining these events or they are secondarily involved, it would be interesting to have a clear map of those factors in order to have a restricted number of potentially “good candidates” for mucosal healing. The present review will present a class of these factors and their modulation in course of therapy, starting from pathogenic studies involving several treatments associated with good clinical outcomes. This approach is meant to help in the difficult task of identifying “good candidates” for healing signatures, which could also be possible new therapeutic targets for clinical management of IBD patients.

## 1. Introduction

Inflammatory bowel diseases (IBDs) are chronic inflammatory conditions characterized by chronic intestinal mucosa damage caused by an abnormal immune response against food or bacterial antigens [1–3]. New therapies, including biologics, have been proved to induce mucosal healing in both Crohn's disease (CD) and ulcerative colitis (UC) [1, 2]. Mucosal healing has been associated with reduced hospitalization, maintenance of remission and better clinical outcomes [4, 5]. 

Mucosal healing in course of IBD is still unclear, starting from its definition. The most commonly known definition of mucosal healing is the “endoscopical” healing, whose quantification has been made possible through endoscopic scores, as Mayo score 7 [6], SES-CD [7], and Rutgeerts' score (especially used to assess the endoscopic recurrence of CD in patients undergone to surgical interventions) [8]. 

Endoscopic healing could be related to an immunological repair of lesions; however a clear dependence of endoscopical healing on “histological healing” has not been found, and furthermore it has not been fully explored the relationship between endoscopy, histology, and biological repair [9]. 

Starting from this hypothesis, many studies tried to find molecular signatures to predict the trend of the disease, searching within the key players of IBD pathogenesis, such as pathways related to mucosal permeability and response to environmental agents [10], genetic factors, as genes involved in intracellular pathogen recognitions (*NOD* [11]), autophagy not mitochondria or chaperone associated (*ATG16L1* [12], *IRGM* [13, 14], or* LRRK2* [15]), cytokines receptor (*IL-23* [11]), or ER stress unfolded protein response elements (*XBP1* [16–18],* AGR2* [19], and *ORMDL3* [20–22]), and genes related to adaptive and innate immune responses.

Products of genes related to adaptive and innate immunity have been extensively associated with injured tissue, where chronic inflammation is sustained by an activation of mast cells/macrophages, neutrophils, and dendritic cells, followed by activation of T cells and other leukocytes. 

Both animal studies and human studies have led to the identification of different subpopulations of T cells that are activated in an aberrant manner, respectively, in CD (Th1 and Th17) [23–25] and UC (Th2) [26]. 

While mucosal healing, and probably “histological healing,” is becoming a reality close related to clinical practice, the “biological healing,” associated with an immunological restoration in gut mucosa, is until applied only in research field, usually in studies assessing the response to a certain therapy in course of IBD. 

Aim of this narrative review is to collect good examples of experimental evidences correlating endoscopic, histological, and biological healing in course of IBD, in response of well-defined therapeutic interventions (Table 1). Therapies will be divided into drugs acting systemically or locally with a broad spectrum of action and systemic drugs with a specific target.

## 2. Systemic or Topical Drugs with Broad Spectrum of Action

### 2.1. 5-Aminosalicylic Acid/Sulfasalazine

Mesazalazine (5-ASA) is one of sulfasalazine derivatives. It is administered starting 2 g/die until 4.8 g/die particularly in UC patients, but also in patients affected by CD [23]. It is produced in tablets, which deliver the active form in colonic mucosa in a pH-dependent or time-dependent way. New formulations of 5-ASA involve a different system able to increase colonic release [24, 25]. Mesalazine also acts locally, being active on rectum and left colon [27]. 5-ASA action depends on its ability to inhibit in vitro leukotriene (LT)B4 and prostaglandin (PG)E2 production. These effects were evaluated in biopsy specimens grown in culture for 24–48 h from healthy and/or UC or CD patients [28]. Other reports show that 5-ASA could decrease IL-1beta production during a 24-hour treatment of biopsy samples from patients with active IBD [29, 30] or inhibit the activation of NF-kB [31], so decreasing the expression of several cytokines (TNF, IL-1, IL-2, IL-6, and IL-8) or adhesion molecules (ICAM-1, VCAM-1, E-selectin, and MAdCAM-1) and enzymes involved in inflammation, like inducible nitric oxide synthase and cyclooxygenase-2 [32, 33]. In 2000, Bantel et al. showed that endoscopic healing in 20 UC patients treated with 5-ASA correlated with a reduced expression of NF-kB at immunohistochemical staining on tissue sections [31]. Elevated levels of LTB4 have been reported in colonic tissue from patients with UC: lipid extracted was analyzed by high-pressure liquid chromatography and biopsy specimens from patients affected by IBD and contained 254 ng of LTB4 per gram, in comparison with mucosa from normal subjects containing less than 5 ng of leukotriene B4 per gram of biopsy weight [34–36]. 5-ASA has shown ability to induce peroxisome proliferator-activated receptor *γ* (PPAR-*γ*) in vitro on HT29 colon epithelial cell line [37]. This result correlates with the finding that PPAR-*γ*, at mRNA and protein levels, is lower on specimens from patients affected by UC compared to CD or controls, and that use of and response to 5-ASA were associated with a reestablishment of its levels [38]. In these patients, the effect was observed only at intestinal mucosal levels and not within peripheral blood mononuclear cells, suggesting that these changes were depending on epigenetic alterations induced by the drug at mucosal level [38]. PPAR-*γ* is a nuclear receptor that activates kinases and other transcription factors implicated in inflammatory process such as nuclear factor kB (NFkB), c-Jun, c-Fos, and nuclear factor of activated T cell (NFAT) [39–41] and inhibits mucosal production of inflammatory cytokines (IL-1*β* and TNF-*α*) and chemokines [42], proliferation of inflammatory cells [43], and expression of some adhesion molecules [44].

### 2.2. Corticosteroids

Steroids are among the most potent anti-inflammatory drugs known in human pharmacology and the most widely bioavailable: their lipophilic characteristics allow corticosteroids to passively diffuse across cellular phospholipid layer and to bind their cytoplasmic receptors expressed in every tissue [45]. The corticosteroid receptor is a member of the nuclear receptor (NR) superfamily, which includes receptors of other hydrophobic molecules like biliary acids, A and D vitamins' and thyroid hormones. NR superfamily shares in common the same structure with three functional domains: at N-terminal part they present a transactivation domain; at C-terminal part there is a ligand-specific binding protein (LBD); between two terminus there is a central zinc finger DNA-binding domain (DBD) which binds specific DNA sequences, termed glucocorticoid-responsive elements (GRE) [46]. This interaction allows the increase of lipocortin 1 syntesis, a phospholipasis A2 inhibitor, inhibition of arachidonic acid release [47], and the increase of IkBa expression which binds NFkB and maintains it inactive [44–46]. Moreover, the heterocomplex corticosteroid-receptor inhibits some interactions among transcriptional factors and their specific genes, like the inhibition of link between NFkB and cytokines sequences [44–46]. Different kinds of steroids are known, and their anti-inflammatory power is usually related to cortisone derivatives [48]. Steroids can act on all cells of our body, particularly immune cells, and that is, probably, the main reason of their efficacy on IBD [49]. Recently, poorly absorbable steroids, active only at mucosal levels, have been shown efficacy in treatment of IBD [50–53]. Because of their structures they are believed to act through the same pathways as systemic steroids, although directly on intestinal mucosa [54]. Active treatment with corticosteroids has reduced activation of NFkB in colonic biopsy of 13 IBD patients as detected by electrophoretic mobility shift assays, following 3 weeks of treatment with 0.75 mg/kg per day prednisolone. [51]. Moreover, some studies also showed a dose-dependent inhibition of intestinal epithelial cell migration and proliferation in bowel, especially prednisolone, budesonide, and dexamethasone at lower concentrations [52, 53, 55]. In vitro studies on intestinal mucosa from IBD patients showed that treatment with dexamethasone lowered levels of IL-1beta and leukotriene B4 [56]. In a recent paper [57] it was shown that in CD patients use of steroids, as well as immune-suppressant and anti-TNF-*α* drugs, was associated with downregulation of MMP-9 and MMP-26 positive neutrophils and stromal TIMP-1 and TIMP-3 and this paralleled histology score and calprotectine. Furthemore, Raddatz et al. analyzed systematically several cytokine mRNA expressions in intestinal mucosa from IBD patients in following oral steroid therapy [58]. IL-1*β*, IL-2, IL-4, IL-6, IL-10, IFN-*γ*, and TNF-*α* were evaluated by quantitative reverse transcriptase-polymerase chain reaction in biopsies and PBMNC, and their changes correlated with endoscopic findings, clinical activity, and outcome after 6 months from therapy. Among all cytokines, IL-1*β*, IL-6, and TNF-*α* were the most represented, but, in contrast to IL-1*β* and TNF-*α*, IL-6 expression was restricted only to inflamed mucosa and correlated better with clinical activity and C-reactive protein levels. Corticosteroids are reported to suppress the levels of cytokine mRNA [59] although they have a paradoxical action of promoting neutrophil survival [60, 61]: therapy with dexamethasone induces eosinophil apoptosis, but it is a great inhibitor of neutrophil apoptosis. Another interesting paper suggested that side effects associated with steroid treatment were associated with an heavy depression of NF-kB activity, which in normal conditions regulates human glucocorticoid (hGC) receptor-l levels in an autoregulative molecular loop [62].

#### 2.2.1. Azathioprine and Cyclosporine

These are among the most used immunosuppressants in IBD. Azathioprin (AZA) therapy was one of the first drugs described to lead to mucosal healing in CD patients [63]. AZA/6-mercaptopurine is an inosin analogue that decreases acid nucleic synthesis especially in lymphocytes, with a decrease of immune responder cells [54]. It was reported that AZA therapy in absence of corticosteroids led to endoscopic mucosal healing in 73% of 19 patients after 6 months [64], while inducing T-cells apoptosis [65]. Moreover, azathioprine is able to maintain mucosal healing in contrast with corticosteroid therapy [63]. Data about treatment of UC with AZA therapy is controversial, especially about the maintenance of remission [66]. In vitro studies analyzing the effects of the drug on T cells from lamina propria of colonic specimens from CD patients, showed that it was able to suppress Rac1 activity genes, like mitogen-activated protein kinase kinase (MEK), NF-kB, and bcl-x(L), leading to a mitochondrial pathway of apoptosis. AZA treatment is associated with disappearance of the inflammatory infiltrate [8] and G2 cell cycle arrest [67]. 

Cyclosporin (CsA) blocks the signaling transduction as it does not allow the calcineurin activation. This is associated with the lack of activation of the transcription factor NFAT and consequently the transcription on IL-2. The CsA pathway is very important in T cells because of their dependency of IL-2 to organize their immune responses [54]. Although there is not a specific effect of CsA on mucosal healing [68], in acute severe UC cyclosporine could be a powerful rescue therapy for patients not responding to steroid treatment [84]. A recent study [85] showed that use of cyclosporine does not avoid colectomy in 50% of subject, as it was not associated with induction of mucosal healing in these patients. 

### 2.3. Systemic Drugs with Specific Targets: Anti-TNF

Recombinant techniques and improvements in molecular biology field allowed to create totally human or humanized monoclonal antibodies, in vitro anti-human cytokines, such as TNF-*α*, and antimembranous proteins like integrins, or phenotype proteins, like anti-*α*4*β*7 antibodies, called “biologic agents.” The production of these molecules allowed to hit specific molecular targets, in order to escape side effects of more wide-spectrum drugs and resulted in a better understanding of the pathophysiology of diseases, and it has been made possible through *phage-display *methods [69].

Drugs actually used in IBD include infliximab, adalimumab, and other molecules still under development or in clinical trials.

#### 2.3.1. Infliximab (IFX)

It is a chimeric monoclonal antiboby, not fully human, with variable regions of Fab with murine origin, directed to human TNF-*α*. It was first released for CD and then for UC therapy. Together with clinical improvement, it has been described that IBD patients receiving IFX showed a decrease of intestinal permeability.

Use of IFX is associated with lowering of microvascular CD40 and VCAM-1 expression in mucosal biopsies evaluated by immunohistochemistry [50]. In the same study the same changes have been showed in serum levels of plasmatic sCD40L and platelet/peripheral blood T-cell (PBT) CD40L expression. 

One of the most important mechanisms of action of IFX is the induction of T-cells apoptosis. It has been shown, in fact, that T cells, isolated by CD3 selection from IBD patients after treatment in vitro with IFX, go to apoptosis [70]. Treatment with IFX was associated with a reestablishment of regulatory T cells (CD4^+^CD25 + Foxp3^+^ T cells or T regs) in intestinal mucosa as well as in peripheral blood, particularly, in responder patients, together with a decrease of their apoptosis [71]. A study (ACCENT I) has demonstrated that mucosal healing occurred in 9 of 10 patients with CD after four weeks of a single infusion of IFX [72]. In this kind of patients an endoscopic substudy on ACCENT I showed mucosal healing in 99 patients: the patients that received 3 infusions of infliximab (0-2-6 weeks) demonstrated mucosal healing compared to the patients that received only 1 infusion of infliximab [86]. A recent study showed the changes of circulating cytokines in CD responder and not-responder patients in IFX treatment during endoscopic evaluation. A significant increase in the plasma TNF-*α* level was found at week 6 in both groups and in contrast, at both week 2 and week 6 the serum IL-6 levels tended to be lower than at baseline [87]. The serum levels of other cytokines (IL-23, IL-17A, and INF-*γ*) did not show significant changes. The authors of the same study hypothesized that the same TNF-*α* stimulated the cytokine production (such as IL-23, IL-12p70, IL-17, and IL-6 syntheses by LPMCs in CD patients [88]) and IFX, blocking TNF-*α*, inhibited their production.

The trials ACT1/2 showed that the use of IFX in patients with UC, already at week 8, was associated with mucosal healing, as indicated by an endoscopic subscore 0-1 compared to baseline at week 0, and that was paralleled to a lower risk of colectomy over the next year [73]. In 2009, an interesting study showed that in patients affected by UC following the induction phase with IFX (5 mg/Kg), TNF-*α* and INF-*γ* mRNA levels in colonic biopsies lowered, but not those of IL-10 and IL-4. Furthermore, decrease in TNF-*α* mRNA was correlated with clinical and endoscopic improvements [74]. 

In another paper assessing 35 CD patients before and after 2 or 6 weeks from starting IFX therapy [87], it was shown that higher levels of IL-17A, IL-23, and IL-12 at baseline were predictive for lower therapeutic response to IFX therapy, as their levels remained high also after therapy.

Biopsies from UC patients treated in vitro with IFX showed that IFX induced a reduction neither in TNF-*α*-mRNA nor of IL-1*β*-mRNA, but of IFN-*γ*-mRNA and, in a lower extent, of IL-6-mRNA [75].

#### 2.3.2. Adalimumab (ADA)

It is a full human antibody against TNF-*α*, licensed for both UC and CD. A prospective study showed how ADA was able to induce endoscopic healing and normalization of mucosal cytokine investigated by mRNA expression in patients with active CD [76]. This study included 77 patients and they were examined by endoscopy before and after therapy (with a minimum of six ADA injections). Biopsies were collected for measurements of mRNA expression levels of IL-17A, IL23, IFN-*γ*, TNF-*α*, IL10, and Foxp3, as well as for immunohistochemistry. Complete endoscopic healing was achieved in 27.3% of patients after 10 weeks of treatment and it was associated with a significant reduction in mRNA expression levels for all cytokines except IL10. Elevated expression of TNF-*α* and IL-17A persisted in 52% and 76%, respectively, of patients with complete endoscopic remission. Pretreatment cytokine gene expression levels did not predict response to ADA therapy. A study about T-cells apoptosis showed ADA was able to induce it increasing the Notch-1 pathway [77]: by immunohistochemical staining, lower levels of Notch-1 were detected in UC inflamed mucosa and it increased in response to anti-TNF*α* treatment. This observation has an important immunological significance as Notch-1 inhibition prevents T-cell cycle arrest (induced by anti-TNF-*α*) but not apoptosis.

## 3. Methodology Used to Assess Immunological Signatures in IBD

As suggested from the above-reported studies, various techniques have been used for different experimental approaches. They can be overall divided into ex vivo studies and in vitro studies and for protein and acid nucleic analyses. The first group, easier to perform, comprehends direct techniques able to characterize immunological signatures on biologic samples fixed in formalin or frozen.For analysis of proteins of outer membrane or cytoplasmic, the most diffused techniques include immunohistochemistry and western blotting, while for nucleic acid analysis, real-time PCR [78], mRNA microarray, and tissue microarray [80]. The greater advantage of microarray is the possibility to screen in the same time several mRNA or protein: cDNA or oligonucleotides are spotted on the slide surface [81, 82]. Tissue microarrays (also TMAs) are paraffin blocks with until 1000 different separate tissue cores: they are assembled in arrays to allow multiplex histological analysis [79, 83]. The great limitation of this tool is that it does not show which part of the tissue is expressing that particular protein. Immunohistochemistry and fluorescent in situ hybridization (FISH), on the contrary, could be complementary to them. Ex vivo study is usually a “static study,” as the above methods report a “picture” of the proteic state of a patient gut mucosa, in a precise moment. Western blot has a good sensitivity, able to discriminate easily a negative compared to a positive result, but it does not allow to localize a specific protein in a tissue, as it starts from protein lysate of the biological specimens like colonic biopsies. 

mRNA analysis by RT-PCR is another very valid method to indirectly evaluate protein levels. It could be used to evaluate a response state of a tissue at different time points.A second group of techniques are dealing with in vitro studies. Many experiments described in this review have been performed by in vitro studies consisting in culturing colonic biopsies or colonic cell lines [29, 89]. The major advantage of these studies is the possibility to work dynamically. Besides methods already described above, this approach allows measurement of released cytokine by ELISA assay, Multiplex assay, and flow cytometry, if they start as membrane proteins [90]. With the last method it is possible to evaluate cellular apoptosis or the nuclear expression of CD4^+^CD25^+^Foxp3^+^ T regulatory cells [91]. Multiprotein ELISA assay is a test that allows to measure up to tens of cytokines at the same time from the same serum or tissue culture supernatants or other biological fluids: it is characterized by a particular technology called Xmap technology [92]. Recently this assay was used to evaluate the serum cytokines profile in UC patients [93]. New approaches, already well utilized in oncobiology research, are the micro-RNAs or miRNAs [94, 95] detection by real time PCR [96, 97]. miRNAs are single-strand oligonucleotides that bind mRNA and disrupt it in cytoplasmatic bodies called P-bodies. This effect inhibits cellular ribosome-associated transduction and protein synthesis. To date, however, specific miRNA controlling production of cytokines is still not completely clarified.

## 4. Conclusions

Endoscopic procedures remain the first line to evaluate response to therapy as well as to assess endoscopic state of the diseases. Mucosal healing has been associated, particularly for studies assessing efficacy of biologic therapy, with amelioration in clinical outcomes like hospitalization or surgery, for both UC and CD. Several studies suggest that mucosal healing or amelioration of mucosal inflammation and clinical outcomes correlate with several changes in mucosal immunity. The majorities of changes registered are related to a reduction in proinflammatory molecules levels or to the reduction in activation of transcription factors such as Nf-KB. A broader effect on mucosal immunity seems to be related to use of steroids, by reduction of several cytokines such as TNF-*α*, IL-1, IL-2, IL-6, and IL-8, for both UC and CD. Use of biologics, particularly IFX, is associated with a modulation of both cytokines expression as well as other important immune components of gut mucosa, such as regulatory T cells. The effect of immunosuppressants, particularly AZA and Csa, is related to their primary effect on immune cells. 5-ASA induces a reduction in integrin expression on mucosal endothelial cells, similarly to what is observed for biologics, particularly IFX. Despite the heterogeneity of different studies reported, different drugs could produce similar results in terms of modulation of selected cytokines or inflammatory pathway. That could probably reflect a common pathway of action of different drugs, or, more likely, the same positive response for a patient, for a therapeutic intervention. These observations open new important consideration on mechanisms of action of different drugs, very often not well known, and secondarily, to the mechanism of healing processes which could share similar pathway between different treatments. When “normal” healing responses are not generated, disequilibrium of cytokine content is observed among patients, that could relate to different and not exhaustive responses to certain drug. These studies open new perspective on discovery of biological and tissue-specific prognostic factors in IBD therapy. As an example, Matsuda et al. showed that patients not responding to therapy were displaying higher levels of TNF-*α* and IL10 in course of UC [98], while higher levels of mucosal TNF-*α* and IL-6 were observed in nonresponding CD [88]. Despite interesting findings, major limits reduced the applicability of these examinations. One of the first reasons is that the majority of studies available are only observational studies. To the best of our knowledge, there are no trials assessing as an endpoint the “biological response,” instead of clinical or endoscopic response or the prospective validity of the mucosal biomarker for both CD and UC. Furthermore, a common, standardized method of analysis used to establish a potential cut-off of reported values is also not available. Finally, whether “old” or classic techniques available are more reliable, widely diffused, and able to assess few targets at the time, new emerging techniques, like microarray analysis or miRNA analysis, display a broader potential to picture the immune and metabolic status of gut mucosa. A broader analysis, however, is more difficult to interpret and mathematic clustering of data still does not correspond to validated or standardized immune-metabolic phenotype, useful for daytime practice. Further studies assessing immune signatures in response to therapy are warmly welcome, particularly those assessing new mechanisms of action of clinical efficacious drugs. This approach could identify good candidates for mucosal prognostic biomarkers, together with new therapeutic targets for future researches.

## Figures and Tables

**Table 1a:** (a) Crohn disease

Treatment	5-ASA	Corticosteroids	AZA; CsA	IFX	ADA
Method	HPLC; ELISA; RT-PCR	ELISA; RT-PCR	ELISA; RT-PCR	ELISA; RT-PCR	ELISA; RT-PCR
Target	Protein; nucleic acid	Protein; nucleic acid	Protein; nucleic acid	Protein; nucleic acid	Protein; nucleic acid
Major findings	↓IL-1beta	↑lipocortin 1	↑T-cells apoptosis	CD40,CD40L, and VCAM-1	=TNF-*α* and IL-17A
	↓TNF-*α*, IL-1, IL-2, IL-6, and IL-8	↑IkBa	↓Rac1	↑T-cells apoptosis	↓IL23, IFN-*γ*, and IL-10
	↓ICAM-1, VCAM-1, E-selectin, and MAdCAM-1	↓NF-*κ*B	↓(MEK), bcl-x(L)	↑CD4 + CD25highFoxp3+ T cells	
	↓NF-*κ*B	↓MMP-9, MMP-26, TIMP-1 and TIMP-3	↓NF-*κ*B	↑Plasma TNF-*α*	
	↑PPAR-g	↓ IL-1*β*, IL-2, IL-4, IL-6, IL-10, IFN-*γ*, TNF-*α*	↑G2 cell cycle arrest	↓SerumIL-6	
	↓LTB4	↓Intestinal epithelial cell migration and proliferation	↓Calcineurin activation	= IL-23, IL-17A, and INF-*γ*	
	↓PGE2	↑Neutrophil survival	↓NFAT		
		↓hGC	↓IL-2		
References	[32, 34–50]	[54–68]	[69–72]	[73–78]	[79]

**Table 1b:** (b) Ulcerative colitis

Treatment	5-ASA	Corticosteroids	AZA; CsA	IFX	ADA
Method	HPLC; ELISA; RT-PCR	ELISA; RT-PCR	ELISA; RT-PCR	ELISA; RT-PCR	IHC;ELISA; RT-PCR
Target	Protein; nucleic acid	Protein; nucleic acid	Protein; nucleic acid	Protein; nucleic acid	Protein; nucleic acid
Major findings	↓IL-1beta	↑lipocortin 1	↑T-cells apoptosis	CD40,CD40L, and VCAM-1	=TNF-*α* and IL-17A
	↓TNF-*α*, IL-1, IL-2, IL-6, and IL-8	↑I*κ*Ba	↑G2 cell cycle arrest	↑T-cells apoptosis	↓IL23, IFN-*γ*, and IL-10
	↓ICAM-1, VCAM-1, E-selectin, and MAdCAM-1	↓NF-kB	↓Calcineurin activation	↑CD4 + CD25highFoxp3 + T cells	↑Notch-1
	↓NF-*κ*B	↓ IL-1*β*, IL-2, IL-4, IL-6, IL-10, IFN-*γ*, and TNF-*α*	↓NFAT	↓TNF-*α* and IFN-g m-RNA	
	↑PPAR-g	↓Intestinal epithelial cell migration and proliferation	↓IL-2	= IL-10 and IL-4 mRNA	
	↓LTB4	↑Neutrophil survival			
	↓PGE2	↓hGC			
References	[32, 34–50]	[54–68]	[69–72]	[80–82]	[79, 83]
